# Enhanced caveolin-1 expression increases migration, anchorage-independent growth and invasion of endometrial adenocarcinoma cells

**DOI:** 10.1186/s12885-015-1477-5

**Published:** 2015-06-10

**Authors:** Natalia Diaz-Valdivia, Denisse Bravo, Hernán Huerta, Soledad Henriquez, Fernando Gabler, Margarita Vega, Carmen Romero, Claudia Calderon, Gareth I. Owen, Lisette Leyton, Andrew F. G. Quest

**Affiliations:** 1 Advanced Center for Chronic Diseases (ACCDiS), Santiago, Chile; 2 Center for Molecular studies of the Cell (CEMC), Programa de Biologia Celular y Molecular, Instituto de Ciencias Biomedicas, Facultad de Medicina, Universidad de Chile, Santiago, Chile; 3 Facultad de Odontología, Universidad de Chile, Santiago, Chile; 4 Facultad de Ciencias Biológicas, Pontificia Universidad Católica de Chile, Santiago, Chile; 5 Departamento de Obstetricia y Ginecologia, Facultad de Medicina, Hospital Clínico de la Universidad de Chile, Santiago, Chile; 6 Laboratorio de Comunicaciones Celulares, Instituto de Ciencias Biomedicas (ICBM). Facultad de Medicina, Universidad de Chile, Av. Indepedencia 1027. Independencia, Santiago, Chile

**Keywords:** Caveolin-1, Endometrial cancer, Proliferation, Migration, Invasion

## Abstract

**Background:**

Caveolin-1 (CAV1) has been implicated both in tumor suppression and progression, whereby the specific role appears to be context dependent. Endometrial cancer is one of the most common malignancies of the female genital tract; however, little is known about the role of CAV1 in this disease.

**Methods:**

Here, we first determined by immunohistochemistry CAV1 protein levels in normal proliferative human endometrium and endometrial tumor samples. Then using two endometrial cancer cell lines (ECC: Ishikawa and Hec-1A) we evaluated mRNA and protein levels of CAV1 by real time qPCR and Western blot analysis, respectively. The role of CAV1 expression in ECC malignancy was further studied by either inducing its expression in endometrial cancer cells with the tumor promotor 12-O-tetradecanoyl-phorbol-13-acetate (4β-TPA) or decreasing expression using short-hairpin RNA constructs, and then evaluating the effects of these changes on ECC proliferation, transmigration, matrigel invasion, and colony formation in soft agar.

**Results:**

Immunohistochemical analysis of endometrial epithelia revealed that substantially higher levels of CAV1 were present in endometrial tumors than the normal proliferative epithelium. Also, in Ishikawa and Hec-1A endometrial cancer cells CAV1 expression was readily detectable. Upon treatment with 4β-TPA CAV1 levels increased and coincided with augmented cell transmigration, matrigel invasion, as well as colony formation in soft agar. Reduction of CAV1 expression using short-hairpin RNA constructs ablated these effects in both cell types whether treated or not with 4β-TPA. Alternatively, CAV1 expression appeared not to modulate significantly proliferation of these cells.

**Conclusion:**

Our study shows that elevated CAV1, observed in patients with endometrial cancer, is linked to enhanced malignancy of endometrial cancer cells, as evidenced by increased migration, invasion and anchorage-independent growth.

**Electronic supplementary material:**

The online version of this article (doi:10.1186/s12885-015-1477-5) contains supplementary material, which is available to authorized users.

## Background

Caveolin-1, a member of the caveolin family of proteins composed of the isoforms caveolin-1, -2 and -3, has been implicated in processes related to cell transformation. Caveolin-1 and -2 are expressed in most tissues, while caveolin-3 distribution is restricted to muscle and glial cells [1, 2]. CAV1 is thought to function as a tumor suppressor in a variety of cellular backgrounds [3–6]. Alternatively, CAV1 is upregulated in some metastatic [6–9] and multidrug resistant cancer cells [6, 10, 11] where expression is associated with cell survival and proliferation [10, 12, 13]. Thus, the role CAV1 plays in tumor development and progression appears to be context dependent.

Endometrial adenocarcinoma is one of the most common malignancies of the female genital tract [14] and is considered a very frequent tumor type in industrialized countries [15]. Generally, little is known about CAV1 in this type of cancer compared with others, like breast cancer [16–20]. In cultured human endometrial cancer (CHEC) cells, increased CAV1 expression is observed during epithelial-mesenchymal transition (EMT) [21]. However, in a microarray study of type I endometrial cancer biopsies, a decrease in CAV1 mRNA was reported when compared with control samples [22]. Thus, whether CAV1 is expressed and how the protein may contribute to the development of endometrial cancer remains an open question.

Phorbol esters are well-known tumor promotors and activators of Protein Kinase Cs (PKCs) that may induce profound alterations in proliferative responses in a cell-type dependent manner [23]. Also effects appear to depend on the degree of tumor progression. In the moderately differentiated endometrial Hec-1A adenocarcinoma cell line, significant increases in proliferation and morphological changes were observed in response to 4β − TPA, while no such changes were reported for the well-differentiated mixed mesodermal cell line SKUT-1-B [24]. On the other hand, 4β − TPA is a well-known activator of PKCs, which in turn are implicated in regulating the expression of CAV1 [25]. However, to our knowledge, no reports are available indicating how 4β − TPA affects CAV1 expression and whether this relates to tumor promotion in endometrial cancer cells (ECC).

Here we examined the possibility that CAV1 might contribute to the transformed phenotype of ECC. We show that CAV1 was expressed at low levels in normal proliferating endometrium, but was readily detectable in endometrial samples of human adenocarcinomas of tumor grades 1-3, as well as in ECC lines. The phorbol ester 4β-TPA was employed as a generic tumor promotor with the objective of establishing how increasing CAV1 expression correlated with malignacy in these cells. Specifically, we demonstrated that 4β − TPA augmented CAV1 expression, as well as enhanced migration, invasive capacity and anchorage-independent growth of two ECC lines. Alternatively, down-regulation of CAV1 using specific short hairpin RNA (shRNA) constructs reduced these characteristics in the same cells. Taken together, these results implicate enhanced CAV1 expression observed in endometrial adenocarcinomas and ECC lines in promoting traits associated with a more malignant and aggressive/invasive cancer phenotype.

## Methods

### Reagents

The following reagents and antibodies were purchased from the sources indicated: 12-O-tetradecanoyl-phorbol-13-acetate (4β-TPA) (Enzo, NY, USA), BCA protein assay kit (Pierce, Rockford, USA), DMEM-F12 (Gibco-BRL, Paisley, UK), Fetal bovine serum (Biological Industries, Kibbutz Beit Haemek, Israel), Histostain-SP Broad Spectrum kit (Invitrogen, Carlsbad, USA), Penicillin, Streptomycin (Gibco-BRL, Paisley, UK), Puromycin (Sigma, USA), Superfect (Qiagen, Valencia, USA), Orthovanadate (Sigma, USA), Antipain, Benzamidine, Leupeptin, Phenylmethyl-sulphonylfluoride (Calbiochem, Germany), EZ-ECL (Biological Industries, Kibbutz Beit Haemek, Israel), RNAase-free DNAase (Promega, Madison, USA), TriZOL (Invitrogen, Carlsbad, USA), M-MLV Reverse Transcriptase (Promega, Madison, USA), Brilliant II SYBR Green Master Mix qPCR kit (Stratagene, USA), MTS® Proliferation Assay Kit (Promega, Madison, USA), low melting point (l.m.p) agarose (Invitrogen, Carlsbad, USA), polyclonal antibodies anti-caveolin-1 (BD, NJ, USA), anti-β-actin and anti-cytokeratin (Sigma, USA), monoclonal antibody anti-caveolin-2 (Santa Cruz Biotechnology, CA, USA), secondary antibody Goat anti-rabbit IgG coupled to horseradish peroxidase (Chemicon-Millipore, Billerica, USA).

### Tissue collection

Normal endometrial samples were obtained with a pipelle suction curette from the corpus of the uteri from healthy women with secondary infertility who attended the Infertility Clinic of the San Borja Arriarán Clinical Hospital, School of Medicine, University of Chile (Santiago, Chile). Biopsies were dated with the patient’s last menstrual period and were histologically confirmed using Noyes criteria [26]. Archival tissue blocks of endometrial tissue were obtained from the Department of Pathology of the San Borja Arriarán Clinical Hospital and utilized for immunohistochemical analysis. Both healthy and pathological subjects had not been exposed to exogenous hormones for at least 3 months and solicited medical assistance due to metrorrhagia, except for those with infertility related problems. All endometrial samples were classified by an experienced histopathologist. This study was approved by the ethics committee of the San Borja Arriarán Clinical Hospital, School of Medicine, University of Chile. All samples were obtained with informed consent of the patients involved.

### Tissue immunohistochemistry

CAV1 was detected by immunostaining of 5-μm sections from formalin-fixed, paraffin-embedded endometrial biopsies. Tissue sections were deparaffinized in xylene and hydrated gradually in graded alcohol. The sections were incubated in antigen retrieval solution (100 mM Tris buffer, pH 9.5) at 100 °C for 20 min. Endogenous peroxidase activity was eliminated by incubating the samples in 0.2 % hydrogen peroxide in methanol for 30 min. Non-specific antibody binding sites were blocked with 4 % PBS–BSA for 1 h. Primary anti-CAV1 antibodies were applied to the samples and incubated overnight at 4 °C in a humidified chamber. Negative controls were analyzed on adjacent sections incubated without primary antibody and using non-immune mouse serum. The biotinylated second antibody was detected using the streptavidin–peroxidase system with 3, 3′diaminobenzidine as the chromogen. Samples were counterstained with hematoxylin. Immunochemical staining for CAV1 was quantified using the Image-Pro Plus v. 4.5.029 software (Media Cybernetics, USA) and the Expression Level Score (ELS) was defined as the Mean Density of CAV1-specific staining/Area.

### Cell culture

Ishikawa cells, originally from a 39-year-old woman, were established as a well-differentiated endometrial adenocarcinoma cell line [27]. Hec-1A cells were established from a 71-year-old woman and are considered a moderately differentiated cell line [28]. Thus, Ishikawa and Hec-1A cells are cell lines characteristic of grade 1 and grade 2 endometrial tumors, respectively [29]. Ishikawa and Hec-1A cells were cultured in DMEM-F12 supplemented with 10 % FBS and antibiotics (10 000 U/ml penicillin, 10 μg/ml streptomycin) at 37 °C, 5 % CO_2_.

### CAV1 shRNA lentiviral infection

Lentiviral transduction particles encoding for shRNA against CAV1 (shCav-1 (#5)) and for luciferase (shLuc) were obtained from the Broad Institute, Cambridge, USA. HEK293T cells were transfected with plasmids for the vector (pLKO.1puro), packaging (Δ8.9–pCMVΔR8.9 and vsv-g–pHCMV-G) and the corresponding shRNA against CAV1 (shCav-1(#5)) and luciferase (shLuc). After 24 h of transfection, using The Superfect® Reagent (Qiagen®, Valencia, USA), supernatants were recovered, aliquoted and stored frozen at -20 °C. The shRNA sequences tested were: CGACGTGGTCAAGATTGACTT (shCav-1-(#3)), GCTTCCTGATTGAGATTCAGT (shCav-1(#5)) and CGCTGAGTACTTCGAA ATGTC (shLuc). ECCs (5×10^5^) were plated and transduced with the indicated shRNAs and selected in puromycin-containing (1 μg/ml) cell culture medium for 1 week.

### Western blotting

Cells were rinsed and harvested in ice-cold PBS containing 1 mM orthovanadate, 10 μg/ml benzamidine, 2 μg/ml antipain, 1 μg/ml leupeptin and 1 mM phenylmethyl-sulphonylfluoride (Ova-BAL-PMSF). Cells were centrifuged at 3000xg for 2 min at 4 °C and the respective cell pellets were lyzed by sonication in extraction buffer (Hepes 20 mM pH 7.4, NP40 0.1 % and SDS 0.1 % plus Ova-BAL-PMSF). The protein concentration of cell extracts was determined using the BCA protein assay kit. Cell extracts were separated by SDS-PAGE, transferred to nitrocellulose, blocked in PBS containing 5 % non-fat milk and probed overnight at 4 °C with the anti-CAV1 antibody (1:5000) diluted in PBS containing 5 % gelatin and 1 % Tween-20. Equal loading per lane was assessed by probing with an anti-β-actin antibody (1:5000). Goat anti-rabbit IgG antibodies coupled to horseradish peroxidase antibodies were used to detect bound first antibodies by EZ-ECL. Protein bands were quantified by densitometric analysis using the ImageJ 1.34 s software (available from NIH at http://imagej.nih.gov/ij/).

### Analysis of mRNA levels by real time RT-PCR

Total RNA was isolated with the reagent TriZOL® following instructions provided by the manufacturer. RNA samples, characterized by electrophoresis in 1 % agarose gels (quality control), were treated with RNAase-free DNAase and employed as templates to generate cDNA using M-MLV Reverse Transcriptase. Oligonucleotides for CAV1 (sense primer 5′– TGGTTTTACCGCTTGCTGTCTG-3′; anti-sense primer 5′– GCAAGTTGATGCGGACA TTGCT-3′) and β-actin (sense primer 5′- TGG CAC CCA GCA CAA TGA AGA -3′; anti-sense primer 5′- GAA GCA TTT GCG GTG GAC GAT -3′) were designed using Mx-Pro – Mx3000P v4.10 software (Stratagene, USA). Real-time PCR was performed using the Stratagene Mx3000p Real-Time PCR System with the Brilliant II SYBR Green Master Mix qPCR kit. The reaction cycle consisted of a first stage for 10 min at 95 °C followed by 40 cycles of consecutive 15-second steps at 95 °C, 60 °C and 72 °C. Data were processed using Stratagene MxPro software, based on the quantification method described by Pfaffl, 2001 [30]. The results are expressed as fold increases in CAV1 mRNA levels.

### Proliferation assay

Ishikawa and Hec-1A cells (3×10^3^) were seeded in 96-well plates and cultured for 24 h in normal medium. Then, cells were serum starved for 24 or 48 h, respectively, prior to 4β-TPA (100 nM) treatment. Cell proliferation was evaluated using the MTS® Proliferation Assay Kit.

### Anchorage independent growth

Ishikawa and Hec-1A cells (1,5×10^3^/well) were suspended in 0,5 mL DMEM-F12 containing 10 % FCS and 0,3 % low melting point (l.m.p) agarose (Invitrogen, Carlsbad, USA). This low percentage agarose was poured on top of a 0,5 mL solidified bottom layer containing 0,8 % l.m.p. agar in a 24-well plate, allowed to solidify at room temperature and then returned to 37 °C. 4β-TPA (100 nM) was added every two days. On days 6-8, plates were photographed and the number of colonies in three random fields was determined. Colony forming efficiency was defined as the number of colonies per total of cells seeded in the fields counted.

### Transwell migration assay

Assays were performed in Boyden Chambers (Transwell Costar, 6.5 mm diameter, 8 μm pore size) according to the manufacturer’s instructions. Briefly, the bottom sides of the inserts were coated with 2 μg/ml fibronectin. 1.5×10^4^ cells were resuspended in serum-free medium and plated on to the top of each chamber insert and serum-free medium was added to the bottom chamber. After 7.5 h, inserts were removed, washed and cells that migrated to the lower side of the inserts were stained with 0.1 % crystal violet in 2 % ethanol and counted in an inverted microscope.

### Matrigel invasion assay

Ishiwaka and Hec-1A cells (6×10^5^) were seeded 24 h before serum deprivation for 24 or 48 h, respectively, and 2×10^5^ cells were seeded over 8 μm-porous inserts covered with matrigel (Matrigel Invasion Chamber 8.0 μm, BD Biosciences, Bedford , USA). 4β-TPA (100 nM) was added for 24 h. The inserts were fixed in cold methanol and presence of cytokeratin was detected by immunocytochemistry using specific antibodies. The membranes were mounted in Mowiol and observed under a light microscope and at least 10 fields were evaluated (40× magnification). The number of cells per field was determined.

### Statistical analysis

All data are expressed as mean ± standard error of mean (SEM) of three indepentent experiments. Data were analyzed using the unparied t-test. All data were processed using INSTAT v. 3.05 (GraphPad Software, San Diego, USA, http://www.graphpad.com). Statistical significance was established at the *P* < 0.05 level.

## Results

### Expression of CAV1 in human endometrial tissues

**Fig. 1:**
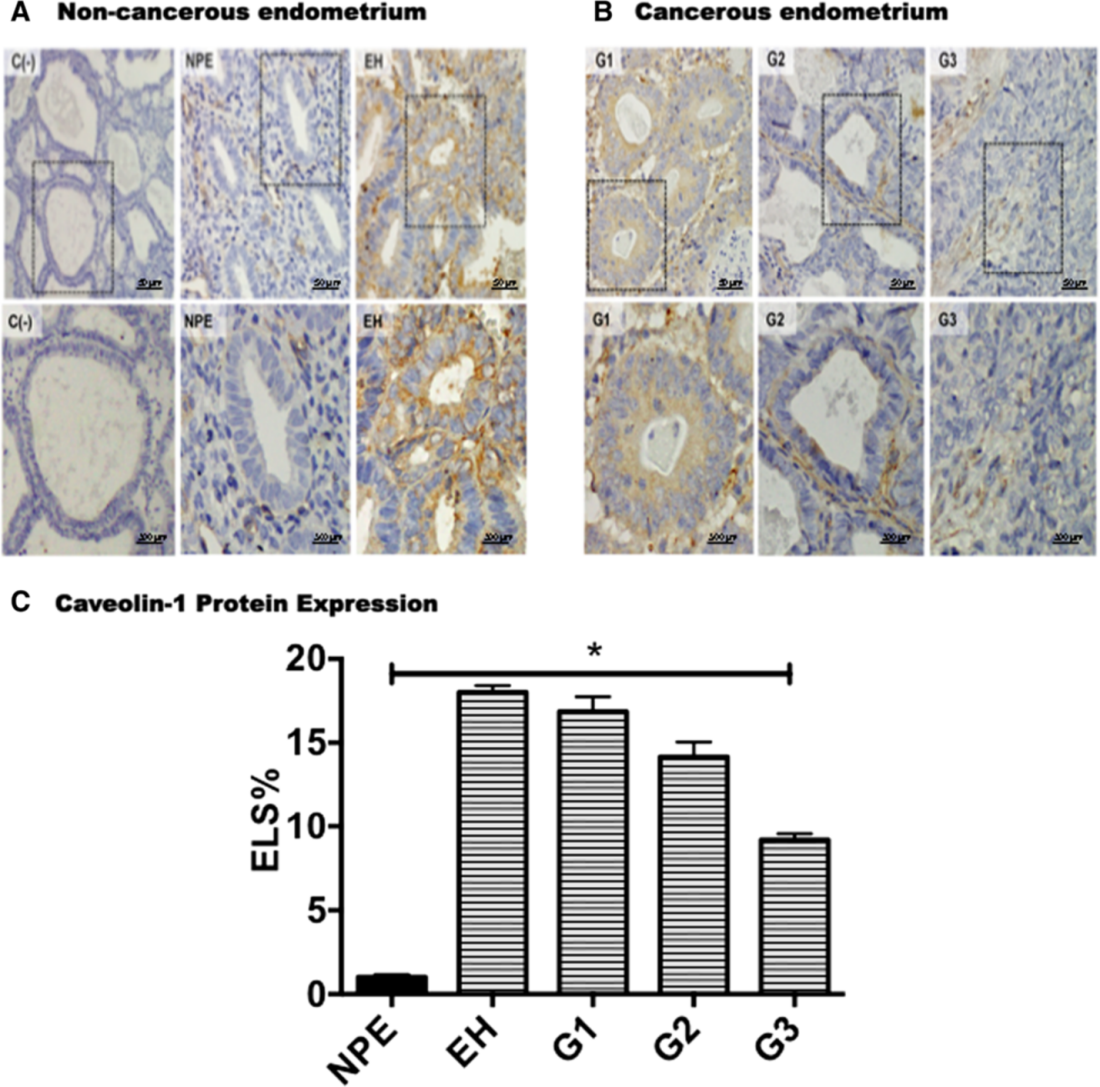
Expression of CAV1 in human normal and pathological endometrial tissues. **a**: CAV1 protein was detected in immunohistochemical sections as described. Representative results shown for non-cancerous endometrium (**a**) include normal proliferative endometrium (NPE, *n* = 3) and endometrial hyperplasia (EH, *n* = 4). A negative control (without primary antibody) from a endometrial tumor grade III sample is included (NC). Results obtained with endometrial tumors grade I (G1, *n* = 3), grade II (G2, *n* = 3) or grade III (G3, *n* = 3) are also shown (**b**). Magnification bar, 50 μm. Boxed areas highlighted in the upper panels are shown at a higher magnification (Magnification bar, 300 μm) in the lower panels. **c**: Specific CAV1 staining was quantified using the ImagePro Plus software (Media Cybernetics, Bethesda, MD, USA). Only epithelial cells were included in this analysis. The Expression Level Score (% ELS) was determined based on averaged optical density and the corresponding area. Statistically significant differences compared to PE using an unpaired t-test are indicated (*, *p* < 0.05; **, *p* < 0.01)

Samples from normal endometrial tissues and endometrial adenocarcinomas were processed for detection of CAV1 by immunohistochemistry (Fig. 1a and b). In normal proliferative endometrium (NPE), CAV1 was barely detectable and predominantly present in the stroma. On the contrary, in hyperplasia, which is characterized by increased proliferation of the endometrial epithelial cells, CAV1 expression was elevated in the glandular epithelium and remained increased in the different stages of endometrial tumor progression (grades 1-3; G1, G2 and G3) compared to NPE (Fig. 1a and b). Quantification and comparison of CAV1 expression in normal and pathological epithelium showed that CAV1 levels were significantly elevated in pre-cancerous stages of hyperplasia, as well as G1, G2 and G3 stages of endometrial adenocarcinoma when compared to NPE (Fig. 1c). Alternatively, while CAV1 expression was readily appreciable in most endometrial tissues, caveolin-2 was barely detectable (data not shown). This result associates elevated caveolin-1 presence in the absence of caveolin-2 with characteristics that may favor tumor progression. This interpretation was subsequently confirmed by observations *in vitro* using endometrial cancer cells (ECC).

### 12-O-tetradecanoyl-phorbol-13-acetate (4β − TPA) increased CAV1 mRNA and protein levels in Ishikawa and Hec-1A cells

**Fig. 2:**
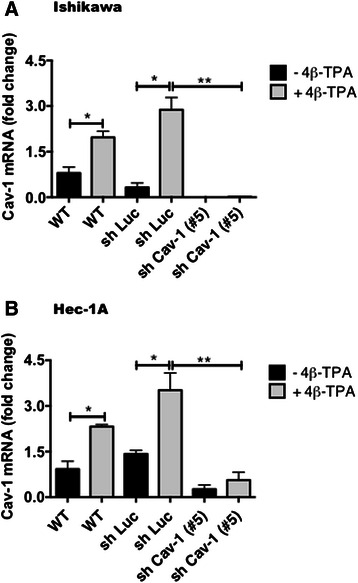
CAV1 mRNA levels increased in ECC after exposure to 4β-TPA. Ishikawa and Hec-1A cells were transduced with CAV1 shRNA (shRNA Cav-1-(#5)) or shRNA for Luciferase (shLuc), as a control. Stably transduced cells expressing the corresponding construct were obtained by selection in medium with puromycin. Wild type or transduced Ishikawa (**a**) and Hec-1A (**b**) cells were seeded in 6-cm dishes for 24 h in complete medium and then cultured in medium without serum for 24 h or 48 h, respectively, prior to 100 nM 4β-TPA stimulation for 24 h. CAV1 mRNA levels were assessed by real time RT-PCR analysis. β-actin was used as an internal control. Values obtained by analysis of three independent experiments are shown for CAV1 mRNA following standardization to β-actin (mean ± SEM). Data were analyzed using the unparied t-test. Statistically significant differences compared with the controls are indicated (*, *p* < 0.05, **,p < 0.01). Note that levels detected for the wild type (WT) cells without 4β-TPA were assigned the reference value 1

**Fig. 3:**
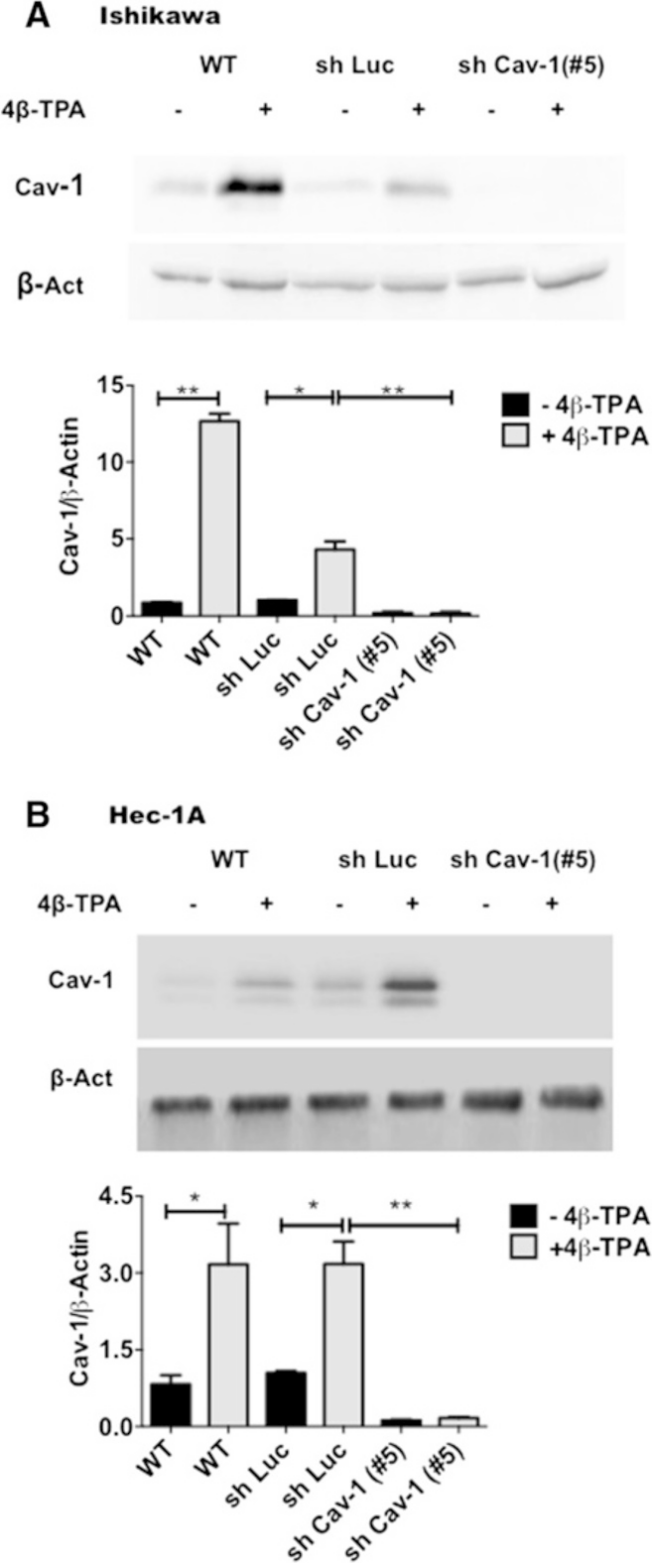
CAV1 expression increased in ECC after exposure to 4β-TPA. Ishikawa and Hec-1A cells were transduced with CAV1 shRNA (shRNA Cav-1-(#5)) or shRNA for Luciferase (shLuc), as a control. Stably transduced cells expressing the corresponding construct were obtained by selection in medium with puromycin. Wild type and transduced Ishikawa (**a**) and Hec-1A (**b**) cells were seeded in 6-cm dishes for 24 h in complete medium and then cultured in medium without serum for 24 h or 48 h, respectively, prior to 100 nM 4β-TPA stimulation for 24 h. CAV1 protein levels were determined by Western blot analysis. β-actin was used as internal control. Values obtained by scanning densitometric analysis of three independent experiments are shown for CAV1 following standardization to β-actin and normalization to shLuc cells without treatment (mean ± SEM). Data were analized using the unpaired t-test. Statistically significant differences compared with the controls are indicated (*, *p* < 0.05, **,p < 0.01). Note that levels detected for the shLuc cells without 4β-TPA were assigned the reference value 1

Expression of CAV1 was analyzed in Ishikawa and Hec-1A cells. Both cell lines expressed CAV1 mRNA and protein, whereby the less differenciated Hec-1A, was found to express more CAV1 mRNA and protein than Ishikawa (data not shown). Thus, CAV1 expression levels correlated inversely with the differentiation status of ECC lines. To study the potential role of this protein in ECC, we first evaluated whether treatment with 4β-TPA, augmented CAV1 levels in Ishikawa and Hec-1A cells (Figs. 2 and 3). Because endogenous CAV1 levels were elevated in both cell lines, we evaluated whether basal CAV1 levels could be decreased by serum starvation. The absence of serum was beneficial for subsequent experiments evaluating migration and invasion, because contributions of proliferation to the results observed were essentially eliminated by this proceedure. Indeed, the changes were greater for Ishikawa and Hec-1A cells after 24 and 48 h of starvation, respectively (Additional file 1: Figure S1). Treatment with 4β − TPA increased CAV1 mRNA in serum-starved Ishikawa (Fig. 2a) and Hec-1A (Fig. 2b) cells compared with non-treated, control cells. Likewise, also CAV1 protein levels increased in 4β − TPA treated Ishikawa and Hec-1A cells (Fig. 3a and b, respectively). Hence, the tumor promoting agent 4β − TPA augmented CAV1 expression in human endometrial adenocarcinoma cells.

In subsequent experiments, CAV1 was down-regulated in ECC by transducing these cells with lentivirus encoding short harpin RNAs specific for CAV1 (shCav-1). As anticipated, CAV1 mRNA and protein levels decreased following lentivirus-mediated transduction, particularly when using the shRNA Cav-1(#5) in both Ishikawa (Figs. 2a and 3a) and Hec-1A (Figs. 2b and 3b) cells. Moreover, 4β-TPA was unable to up-regulate CAV1 in shRNA Cav-1(#5) Ishikawa or Hec-1A cells (Figs. 2 and 3). Finally, in Ishikawa and Hec-1A cells transfected with the shLuc control construct, slightly elevated basal CAV1 levels were observed. Nonetheless, addition of 4β-TPA increased CAV1 mRNA and protein levels significantly (Figs. 2 and 3). Here, it should be noted that similar results were obtained with an alternative short hairpin construct, shRNA Cav-1(#3), although efficiency was lower (Additional file 2: Figure S2). For this reason, shRNA Cav-1(#5) and shLuc control cells were compared in subsequent experiments.

### Alterations in proliferation following down-regulation of CAV1 in ECC

**Fig. 4:**
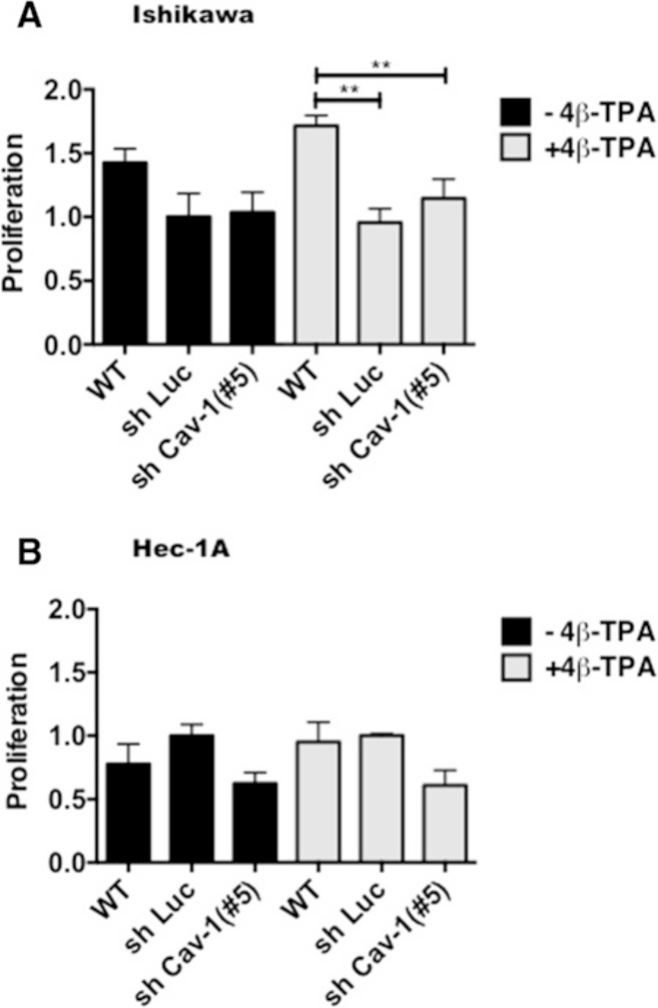
Effects of CAV1 down-regulation on ECC proliferation. Wild type, shLuc and shCav-1(#5) Ishikawa (**a**) and Hec-1A (**b**) cells were seeded in complete medium in 96-well plates 24 h prior to serum withdrawal for an additional 24 or 48 h of culture, respectively. After 24 h of 100 nM 4β-TPA treatment, cell proliferation was evaluated using the MTS® assay according to the manufacturer’s instructions. Data averaged from three independent experiments are shown (mean ± SEM). Statistically significant differences compared with the control group (shLuc cells without 4β-TPA, reference value 1 ) are indicated (*, *p* < 0.05)

Because CAV1 was suggested to favor cell proliferation in breast cancer cells [13], we evaluated whether alterations in CAV1 levels affected proliferation in Ishikawa and Hec-1A cells transduced with the shCav-1(#5) or shLuc contructs. First, shLuc and shCav-1(#5) Ishikawa or Hec-1A cells were seeded in the presence of serum for 24 h and then serum-starved for 24 or 48 h, respectively, prior to evaluating cell proliferation. No differences in proliferation were detectable between shCav-1(#5) tranduced Ishikawa cells compared to the control shLuc cells in the absence of 4β-TPA, although a trend towards reduced proliferation was apparent; however, in the presence of 4β-TPA the previously detected trend became signifcant (Fig. 4a). For shCav-1(#5) Hec-1A cells, a tendency towards decreased proliferation was observed compared with the control shLuc cells both in the absence and presence of 4β-TPA (Fig. 4b), although this trend was never statistically significant. Taken together, these results suggest that CAV1 presence in the absence of 4β-TPA does not modulate ECC proliferation. Here, it should be noted that 4β-TPA was also unable to stimulate significantly proliferation of wild-type cells (Fig. 4a and b).

### CAV1 expression in ECC favored anchorage-independent growth

**Fig. 5:**
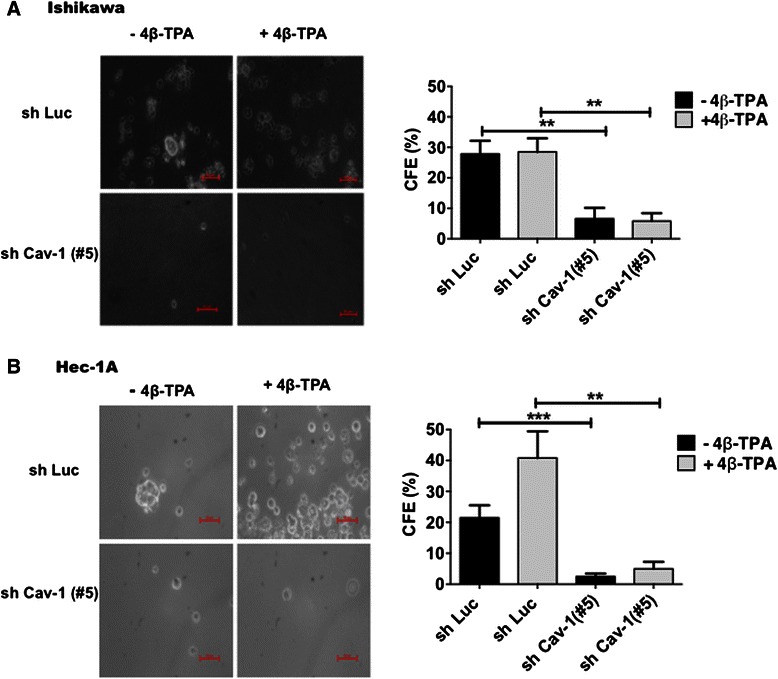
CAV1 expression in ECC favored anchorage independent growth. Anchorage-independent growth was examined for shLuc or shCav-1(#5) Ishikawa and Hec-1A cells in soft agar. Colony-forming efficiency (CFE) was determined as the number of colonies per total number of visible cells. Statistically significant differences compared with the corresponding control group are indicated (**, *p* < 0.01, ***, p < 0.001). Ishikawa (**a**) and Hec-1A (**b**) cells were treated with either 100 nM 4β-TPA in DMEM-F12 in the absence of serum for 6-8 days. shLuc Ishikawa or Hec-1A cells without 4β-TPA were considered as the control groups (28 ± 4 or 22 ± 4 colonies, respectively)

Anchorage-independent growth *in vitro* in soft agar is thought to reflect the capacity of cells to establish tumors *in vivo*. Hence, the ability of 4β-TPA to promote colony formation in soft agar of Ishikawa and Hec-1A cells, transduced with either shLuc or shRNA Cav-1(#5), was tested (Fig. 5). In the absence of 4β-TPA, shLuc Ishikawa and Hec-1A cells formed colonies and this ability was reduced in shRNA Cav-1(#5) cells (Fig. 5a and b, respectively). Interestingly, the presence of 4β-TPA slightly enhanced colony formation only in shLuc Hec-1A, but not in shLuc Ishikawa cells. However, shRNA Cav-1(#5) cells decreased colony formation efficiency in the two cell lines, both in the absence or presence of 4β-TPA, thereby implicating CAV1 in promoting anchorage-independent growth in ECC.

### CAV1 expression in ECC augmented transmigraton and invasion

**Fig. 6:**
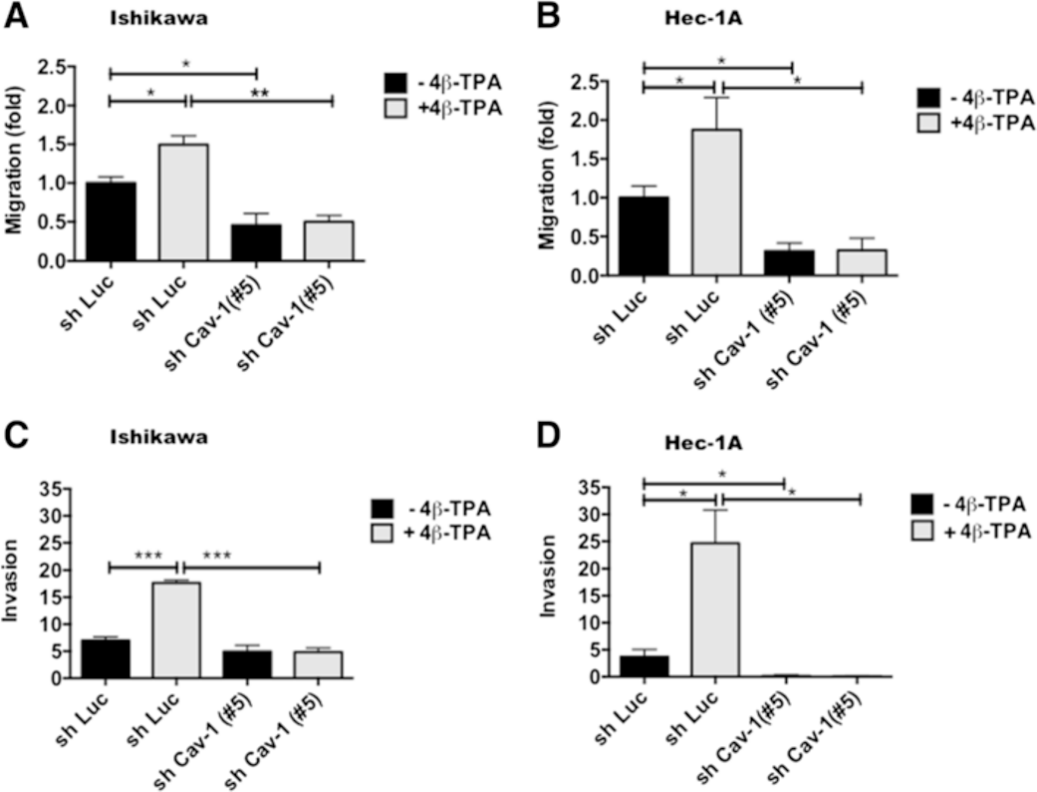
CAV1 expression in ECC augmented transmigration and invasion. To evaluate the role of the up-regulation of CAV1 mediated by 4β-TPA in transmigration 6 × 10^5^ shLuc and shCav-1(#5) Ishikawa or Hec-1A cells were seeded in 6 cm plates in complete medium for 24 h prior to serum withdrawal for an additional 24 or 48 h of culture, respectively. After 24 h of 100 nM 4β-TPA treatment 2 × 10^5^ shLuc and shCav-1(#5) Ishikawa (**a**) or Hec-1A (**b**) cells were seeded in Boyden Chambers coated on the lower side with fibronectin (2 μg/ml) and allowed to migrate in the absence of serum for 7.5 h. The cells that migrated through the pores and bound to the lower fibronectin-coated surface were stained and counted. Values obtained were normalized to the shLuc cells without treatment. The number of transmigrated shLuc cells observed in panels **a** and **b** were 48 ± 9 and 94 ± 26, respectively. Averages from three independent experiments are shown (mean ± SEM). Statistically significant differences compared with the corresponding control group are indicated (*, *p* < 0.05). To evaluate the role of the up-regulation of CAV1 mediated by 4β-TPA in invasion, shLuc and shCav-1(#5) Ishikawa and Hec-1A cells were seeded in 6 cm plates in complete medium 24 h prior serum withdrawal for an additional 24 or 48 h of culture, respectively. After 24 h of 100 nM 4β-TPA treatment 2 × 10^5^ shLuc and shCav-1(#5) Ishikawa (**c**) and Hec-1A (**d**) cells were seeded over matrigel covered porous inserts, with or without 100 nM 4β-TPA for 24 h. The number of cells that invaded the matrigel and migrated through the pores were determined after immunostaining for cytokeratin. Averages from three independent experiments are shown (mean ± SEM). Statistically significant differences compared with the corresponding control group are indicated (*, *p* < 0.05, **, p < 0.01, ***, p < 0.001)

Because the ability to migrate and permeate the extracellular matrix are important events in cancer metastasis, we then evaluated the effect of 4β-TPA-mediated up-regulation of CAV1 in transmigration assays in the absence of serum. To that end, the transduced Ishikawa and Hec-1A cells were cultured without serum 24 or 48 h prior to treatment with 100 nM 4β-TPA. As expected, migration was substantially increased after 4β-TPA stimulation in shLuc Ishikawa and shLuc Hec-1A cells in comparison with the non-treated cells (Figs. 6a and b). Moreover, this effect was not seen in shRNA Cav-1(#5) Ishikawa and Hec-1A cells (Figs. 6a and b). It should be noted again that similar results to those reported here for shCav-1(#5) were obtained with shCav-1(#3) cells (Supplementary Fig. 2c and d), in which CAV1 knock-down was not as efficient (Supplementary Fig. 2a and b). Thus, loss of CAV1 significantly reduced the migration of both adenocarcinoma cell lines in the absence or presence of 4β-TPA.

### Down-regulation of CAV1 decreased matrigel invasion of ECC

To assess whether increased CAV1 expression was linked to invasion, the shLuc and shCav-1(#5) Ishikawa and Hec-1A cells were evaluated in matrigel assays (Fig. 6c and d, respectively). We observed that 4β-TPA significantly enhanced the invasiveness of shLuc Ishikawa and Hec-1A cells and that the invasive potential was substantially reduced in cells expressing shRNA Cav-1(#5). Thus, CAV1 expression in ECC is associated with enhanced invasiveness in matrigel assays.

Taken together our results associate basal, as well as 4β-TPA-enhanced CAV1 expression in ECC with enhanced malignancy, as defined by increased anchorage-independent growth, transmigration and matrigel invasion.

## Discussion and conclusions

CAV1 is known to be upregulated in later stages of many cancers, to enhance migration and invasion of tumor cells of different origin, and to be associated with poor patient prognosis. Whether this may be the case for ECCs has not been determined. Here we show that CAV1 was detected in cancerous epithelial cells of the human endometrium at substantially elevated levels compared with the normal proliferating epithelium. In the human ECC lines Ishikawa and Hec-1A CAV1 was also present. In addition, in both Ishikawa and Hec-1A cells, exposure to the tumor promoter 4β − TPA increased CAV1 protein and mRNA levels. Importantly, reduction of endogenous CAV1 expression using specific shRNA constructs diminished ECC migration, invasion and anchorage-independent growth. Thus, CAV1 expression is associated with enhanced maligancy of ECCs.

Several studies have shown that CAV1 expression decreases during the genesis of human tumors in tissues such as lung, breast, colon and ovary. Additionally, re-expression of CAV1 is sufficient to revert the transformed phenotype of mammary [31], colon [6] and ovarian carcinoma cells [32]. These studies suggest that CAV1 may function as a tumor suppressor protein, possibly by inhibiting signaling pathways aberrantly activated in cancer cells and promoting cell death [25, 32].

Alternatively, CAV1 also promotes malignant characteristics in cancer cells, like anchorage-independent growth [33], cell proliferation [13], multi-drug resistance [16, 17], cell polarization and migration [34–36] and metastasis [8, 37]. Thus, the frequently observed initial decrease in CAV1 expression appears to be a reversible process, whereby increased expression of CAV1 at later stages is then associated with elevated metastatic potential in colon [9], lung [38] and prostate cancer cells [39] and with poor prognosis for prostate [7] or colon [9] cancer patients. Taken together, these data indicate that CAV1 function may change rather dramatically during tumor progression [40, 41]. Interestingly, however, the current study points towards a unique pattern of CAV1 expression in the human endometrium, because CAV1 levels were consistently elevated in all pathological stages. Thus, although in this tissue elevated CAV1 expression appears relevant to tumor development, it cannot *per se* be associated with tumor progression in endometrial cancer patients and additional factors must be invoked to explain how CAV1 presence may favor traits associated with a more aggressive phenotype in ECC.

In agreement with this more complex role, CAV1 mRNA and protein levels reportedly increase with the passage number of CHEC cells and morphological changes characteristic of EMT [21]. However, in the same study CAV1 expression in the highly differentiated Ishikawa cell line was significantly lower than in CHEC cells derived from a stage I grade III endometrial adenocarcinoma, underscoring that the relationship between CAV1 expression and tumor progression is complex. Indeed, our results corroborate this notion, because CAV1 presence enhanced malignancy of ECC, yet protein levels were lower in Ishikawa than in the moderately differentiated Hec-1A cells.

Phorbol esters like 4β-TPA are known activators of PKCs that promote tumor formation in two stage cancer models in mice [23]. Although, they have been shown to increase CAV1 expression in human lung fibroblast cells [42], the possibility that phorbol ester induced tumor promotion may be linked to changes in CAV1 has not been previously considered. Here we observed in ECC that 4β-TPA treatment increased CAV1 expression. Moreover, elevated CAV1 levels in these cells augmented invasive capacity/migration and anchorage-independent growth *in vitro*, characteristics associated with more aggressive tumor cell behavior *in vivo*. Hence, CAV1 presence in ECC favors the malignant tumor cell phenotype and elevated expression in response to phorbol esters is associated with tumor promotion in these cells.

Here it should be noted that 4β − TPA triggers inflammatory responses by increasing the expression of COX-2 through PKCα dependent pathways [43]. Interestingly, our preliminary immuno-histochemical analysis identified substantial levels of PKCα both in normal and diseased tissue samples (data not shown). However, levels did not vary as a consequence of disease (data not shown). Clearly, further experiments are required to determine to what extent PKCα is involved in regulating CAV1 expression. This possibility merits further consideration, given that both PKCα and CAV1 have been independently associated with increased proliferation/survival and migration/invasion phenotypes in certain cancer cells [44, 45]

In summary, this study analyzes for the first time CAV1 expression in the normal endometrial epithelium and adenocarcinoma cells. Basal CAV1 expression, as well as tumor promotor 4β − TPA induced CAV1 up-regulation in endometrial adenocarcinoma cells were linked to enhanced ECC malignancy. Thus, while elevated CAV1 levels *per se* cannot be interpreted as a marker of tumor progression because no significant differences in protein levels were detected when comparing tissue samples from stages I, II and III, the presence of this protein in ECC clearly favored traits associated with a more malignant phenotype.

## Additional files

Additional file 1: Figure S1. CAV1 protein levels in ECC after treatment with 4β-TPA in the presence or absence of serum. Ishikawa (A) and Hec-1A (B) cells were seeded in 6-cm dishes for 24 h in complete medium and then cultured in the absence or presence of 4β-TPA (100 nM) (Lanes 1 and 2, respectively) or were cultured in medium without serum for 24 h (Ishikawa) or 48 h (Hec-1A) and subsequently in the presence or absence of 4β-TPA (Lanes 3 and 4, respectively). CAV1 protein levels were determined by Western blot analysis. β-Actin was used as an internal control.
Additional file 2: Figure S2. CAV1 silencing in ECC inhibits 4β-TPA-enhanced cell migration. Ishikawa and Hec-1A cells were transduced with CAV1 shRNA (shRNA Cav-1(#3) or shRNAfor luciferase (shLuc), as a control. Stably transduced cells expressing the corresponding construct were obtained by selection in medium with puromycin. Transduced Ishikawa (A) and Hec-1A (B) cells were seeded in 6-cm dishes for 24 h in complete medium and then cultured in medium without serum for 24 h or 48 h, respectively, prior to 4β-TPA (100 nM) stimulation for 24 h. CAV1 protein levels were determined by Western blot analysis. β-Actin was used as an internal control. 6×10^5^ shLuc and shCav-1(#3) Ishikawa or Hec-1A cells were seeded in 6-cm plates in complete medium for 24 h prior to serum withdrawal for an additional 24 or 48 h of culture, respectively. After 24 h of treatment with 4β-TPA (100 nM) , 2×10^5^ shLuc and shCav-1(#3) Ishikawa (C) or Hec-1A (D) cells were seeded in Boyden chambers coated with fibronectin (2 μg/ml) and allowed to migrate in the absence of serum for 7.5 h. The cells that migrated through the pores were stained and counted. Values obtained were normalized to the shLuc cells without treatment. Data averaged from two independent experiments are shown.

## Abbreviations

ECC: Endometrial cancer cells
4β-TPA: 12-O-tetradecanoyl-phorbol-13-acetate
shCav-1: Short hairpin RNA against *caveolin-1*
shLuc: Short hairpin RNA against *luciferase*
WT: Wild type
