# Supplementary figures and images for: Enhanced caveolin-1 expression increases migration, anchorage-independent growth and invasion of endometrial adenocarcinoma cells
Source: BMC Cancer. 2015 Jun 10;15:463. doi: 10.1186/s12885-015-1477-5 (PMC4460862; doi:10.1186/s12885-015-1477-5)

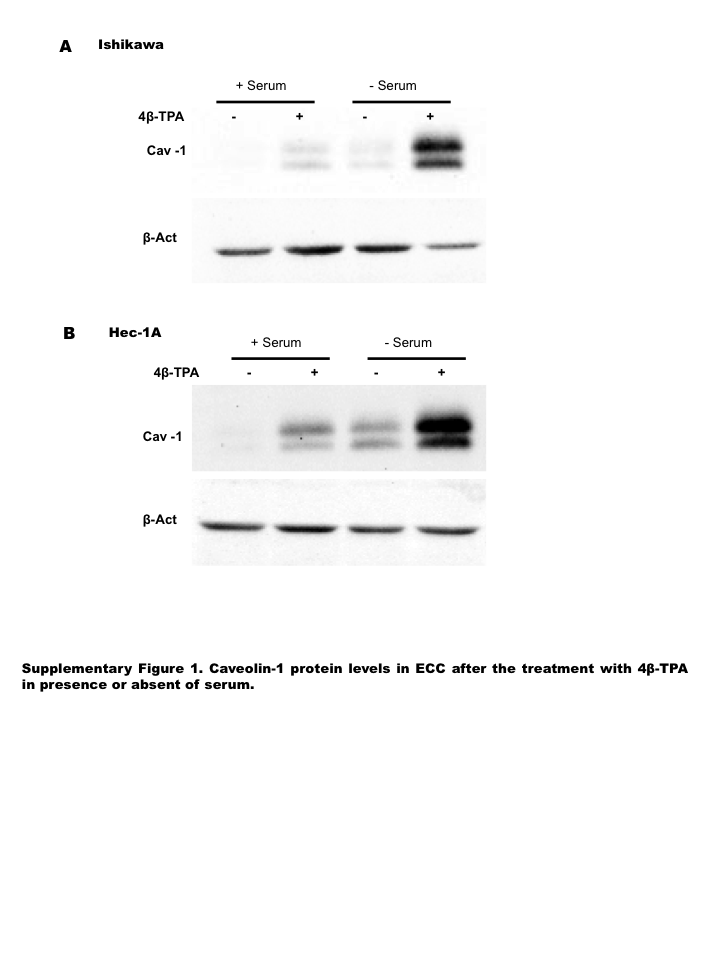

Supplement: Additional file 1: Figure S1. — CAV1 protein levels in ECC after treatment with 4β-TPA in the presence or absence of serum. Ishikawa (A) and Hec-1A (B) cells were seeded in 6-cm dishes for 24 h in complete medium and then cultured in the absence or presence of 4β-TPA (100 nM) (Lanes 1 and 2, respectively) or were cultured in medium without serum for 24 h (Ishikawa) or 48 h (Hec-1A) and subsequently in the presence or absence of 4β-TPA (Lanes 3 and 4, respectively). CAV1 protein levels were determined by Western blot analysis. β-Actin was used as an internal control. [file 12885_2015_1477_MOESM1_ESM.tiff]

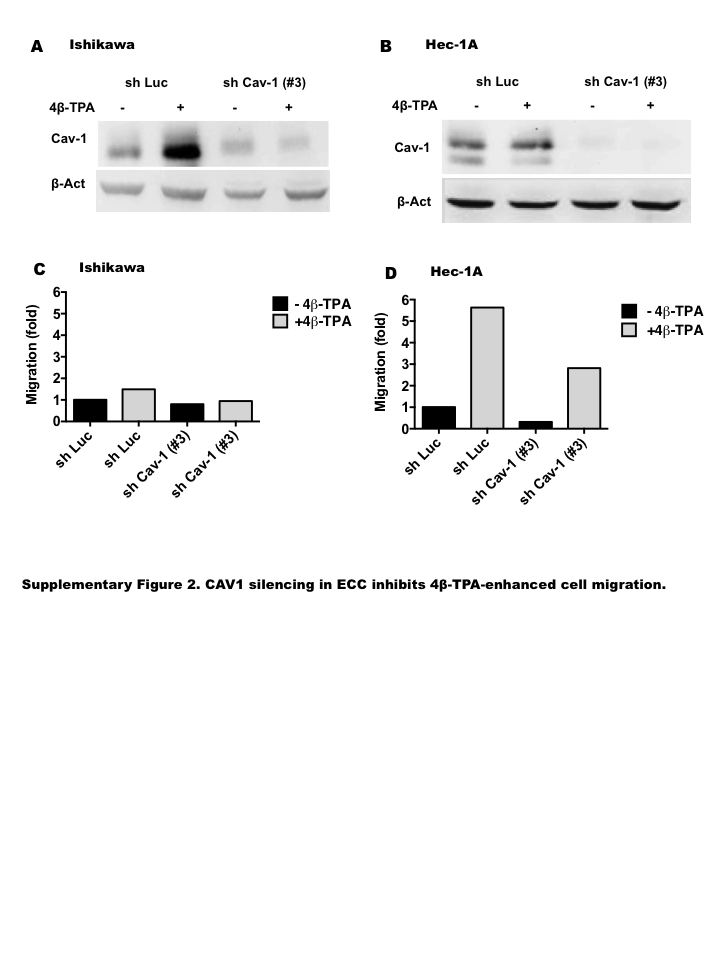

Supplement: Additional file 2: Figure S2. — CAV1 silencing in ECC inhibits 4β-TPA-enhanced cell migration. Ishikawa and Hec-1A cells were transduced with CAV1 shRNA (shRNA Cav-1(#3) or shRNAfor luciferase (shLuc), as a control. Stably transduced cells expressing the corresponding construct were obtained by selection in medium with puromycin. Transduced Ishikawa (A) and Hec-1A (B) cells were seeded in 6-cm dishes for 24 h in complete medium and then cultured in medium without serum for 24 h or 48 h, respectively, prior to 4β-TPA (100 nM) stimulation for 24 h. CAV1 protein levels were determined by Western blot analysis. β-Actin was used as an internal control. 6×10^5^ shLuc and shCav-1(#3) Ishikawa or Hec-1A cells were seeded in 6-cm plates in complete medium for 24 h prior to serum withdrawal for an additional 24 or 48 h of culture, respectively. After 24 h of treatment with 4β-TPA (100 nM) , 2×10^5^ shLuc and shCav-1(#3) Ishikawa (C) or Hec-1A (D) cells were seeded in Boyden chambers coated with fibronectin (2 μg/ml) and allowed to migrate in the absence of serum for 7.5 h. The cells that migrated through the pores were stained and counted. Values obtained were normalized to the shLuc cells without treatment. Data averaged from two independent experiments are shown. [file 12885_2015_1477_MOESM2_ESM.tiff]
